# The Rare Anaphylaxis-Associated FcγRIIa3 Exhibits Distinct Characteristics From the Canonical FcγRIIa1

**DOI:** 10.3389/fimmu.2018.01809

**Published:** 2018-08-20

**Authors:** Jessica C. Anania, Halina M. Trist, Catherine S. Palmer, Peck Szee Tan, Betty P. Kouskousis, Alicia M. Chenoweth, Stephen J. Kent, Graham A. Mackay, Alberta Hoi, Rachel Koelmeyer, Charlotte Slade, Vanessa L. Bryant, Philip D. Hodgkin, Pei Mun Aui, Menno C. van Zelm, Bruce D. Wines, P. Mark Hogarth

**Affiliations:** ^1^Immune Therapies Group, Burnet Institute, Melbourne, VIC, Australia; ^2^Department of Immunology and Pathology, Central Clinical School, Monash University, Melbourne, VIC, Australia; ^3^Monash Micro Imaging, Monash University, Clayton, VIC, Australia; ^4^Department of Microbiology and Immunology, University of Melbourne, Parkville, VIC, Australia; ^5^Melbourne Sexual Health Centre, Central Clinical School, Monash University, Melbourne, VIC, Australia; ^6^ARC Centre of Excellence in Convergent Bio-Nano Science and Technology, University of Melbourne, Parkville, VIC, Australia; ^7^Department of Pharmacology & Therapeutics, The University of Melbourne, Parkville, VIC, Australia; ^8^Department of Medicine, Monash Medical Centre, Clayton, VIC, Australia; ^9^Department of Medical Biology, The University of Melbourne, Parkville, VIC, Australia; ^10^Walter and Eliza Hall Institute for Medical Research, Royal Melbourne Hospital, Parkville, VIC, Australia; ^11^Department of Clinical Immunology and Allergy, Royal Melbourne Hospital, Parkville, VIC, Australia; ^12^The Jeffrey Modell Diagnostic and Research Centre for Primary Immunodeficiencies, Melbourne, VIC, Australia; ^13^Department of Pathology, The University of Melbourne, Parkville, VIC, Australia

**Keywords:** Fc receptors, common variable immunodeficiency, immunodeficiency, systemic lupus erythematosus, immune complex, non-human primates

## Abstract

FcγRIIa is an activating FcγR, unique to humans and non-human primates. It induces antibody-dependent proinflammatory responses and exists predominantly as FcγRIIa1. A unique splice variant, we designated FcγRIIa3, has been reported to be associated with anaphylactic reactions to intravenous immunoglobulins (IVIg) therapy. We aim to define the functional consequences of this FcγRIIa variant associated with adverse responses to IVIg therapy and evaluate the frequency of associated SNPs. FcγRIIa forms from macaque and human PBMCs were investigated for IgG-subclass specificity, biochemistry, membrane localization, and functional activity. Disease-associated SNPs were analyzed by sequencing genomic DNA from 224 individuals with immunodeficiency or autoimmune disease. FcγRIIa3 was identified in macaque and human PBMC. The FcγRIIa3 is distinguished from the canonical FcγRIIa1 by a unique 19-amino acid cytoplasmic insertion and these two FcγRIIa forms responded distinctly to antibody ligation. Whereas FcγRIIa1 was rapidly internalized, FcγRIIa3 was retained longer at the membrane, inducing greater calcium mobilization and cell degranulation. Four *FCGR2A* SNPs were identified including the previously reported intronic SNP associated with anaphylaxis, but in only 1 of 224 individuals. The unique cytoplasmic element of FcγRIIa3 delays internalization and is associated with enhanced cellular activation. The frequency of the immunodeficiency-associated SNP varies between disease populations but interestingly occurred at a lower frequency than previously reported. None-the-less enhanced FcγRIIa3 function may promote a proinflammatory environment and predispose to pathological inflammatory responses.

## Introduction

Receptors for the Fc portion of IgG (FcγR) play major roles in the action of antibodies *in vivo*, including the development of pathological, pro-inflammatory responses in a number of inflammatory diseases [reviewed in Ref. (1–4)]. The balance of activating and regulatory signals from FcγR is critical for appropriate responses that avoid pathological inflammation. The low affinity receptor, FcγRIIa which avidly binds IgG complexes, is only found in human and non-human primates (NHP) (3) and is associated with protective immunity [reviewed in Ref. (1–4)] but is also involved in the development of destructive inflammation (1, 5, 6). Several disease-associated alleles have been identified, in particular the high/low-responder FcγRIIa polymorphism (7, 8) which have altered specificity for IgG and are associated with auto-inflammatory disease (5, 6, 9–14).

FcγRIIa is also unusual as it is the only receptor where the ligand binding chain contains an immunoreceptor tyrosine activation motif (ITAM). Aggregation of FcγRIIa by IgG immune complexes induces tyrosine phosphorylation of its ITAM, initiating a signaling cascade including intracellular calcium mobilization and ultimately pro-inflammatory cell responses [reviewed in Ref. (3, 15)].

Three RNA splice variants of the *FCGR2A* gene have been defined: the canonical FcγRIIa1 is well characterized and is the most widely expressed FcγR being present on platelets and all leukocytes with the exception of lymphocytes (2, 16). FcγRIIa2 mRNA encodes a variant of uncertain physiological significance that lacks a hydrophobic segment of the transmembrane exonic sequence (17, 18). More recently a cell surface variant, FcγRIIa^exon6*^, was identified in common variable immunodeficiency (CVID) patients with adverse reactions to treatment with intravenous immunoglobulins (IVIg), however, further investigation is required to understand the mechanism behind this (19).

We have identified an isoform of FcγRIIa in human and NHP (Pig-tail macaque; *Macaca nemestrina*). This variant, which we designate as FcγRIIa3, is present in both species and is identical to the canonical FcγRIIa1 with the exception of a 19-amino acid insertion in the juxta-membrane region of the cytoplasmic tail in FcγRIIa3. This insert in FcγRIIa3, is related to a similar cytoplasmic segment of the inhibitory Fc receptor, FcγRIIb1 that affects cellular localization (20–22).

The human FcγRIIa3 is identical to the reported FcγRIIa^exon 6*^, arising from a *FCGR2A^c742+871A^*^>^*^G^* SNP (19) which is associated with anaphylactic responses to IgG replacement therapy. We show that longer FcγRIIa3 retention at the cell membrane in comparison to FcγRIIa1 increased signaling. This intronic SNP we found was less frequent in our patients being found in only 1 of 224 individuals, and not present in our CVID nor systemic lupus erythematosus (SLE) patients.

## Materials and Methods

### Animals

Peripheral blood from outbred 3- to 5-year-old pig-tailed macaques (*M. nemestrina*) was obtained from the Australian National Non-Human Primate Facility and studies were approved by the University of Melbourne and Commonwealth Scientific and Industrial Research Organization Animal Health Institutional Animal Ethics Committees. Whole venous blood was obtained from animals sedated with ketamine, as previously described (23), and PBMCs were isolated over Ficoll-Hypaque (GE Healthcare).

### Human Donors

All studies were conducted according to the Declaration of Helsinki principles. Blood samples were collected following written informed consent obtained from 224 volunteers including 55 healthy donors defined as not having a medical history of hematological nor immunological disease. Seventy-eight immunodeficient patients comprised 46 with CVID; 15 patients with hypogammaglobulinemia or specific antibody or IgG-subclass deficiency; 11 with selective IgA-deficiency (sIgAD) and 6 X-linked agammaglobulinemia (XLA) patients. Ethics approval was obtained from local administering institutions: Monash University (2015-0344; 2016-0289), Alfred Health (497/11 and 109/15), Melbourne Health (2009.162), and Walter and Eliza Hall Institute (WEHI, 10/02). No anaphylactic responses were recorded in any patient receiving IgG replacement therapy.

SLE patient mRNA was obtained from 91 patients from the Australian Lupus Registry (ALR) under Monash University Human Research Ethics approval 14262A and 15510L. The ALR and Biobank is a longitudinal study of patients fulfilling at least 4 out of 11 American College of Rheumatology classification criteria or satisfied the new SLE International Collaborating Clinics Classification criteria. Disease manifestations and co-morbidities are characterized at enrollment.

### Isolation of FcγR mRNA

Total RNA was isolated (RNeasy Mini Kit, Qiagen) and cDNA produced from macaque and human PBMCs or sorted CD14+ monocytes (AffinityScript quantitative cDNA synthesis kit, Agilent Technologies). Previously published primers (24) were used to generate FcγR PCR fragments (Sigma-Aldrich) and sequences determined (Big Dye version 3.1 terminator cycle sequencing, Applied Biosystems). All cDNA sequences have been submitted to GenBank (submission no. 1976299).

### Expression of FcγRII Isoforms in Transfected Cells

FcγR DNA was introduced into FcR-deficient IIA1.6 cells using a pMXI retroviral expression system as described (24). The EGFP tagged receptors were generated as follows. FcγRIIa1 [clone Hu3.0 (25)] C-terminus was fused to the N-terminus of EGFP (pEGFP-N1). The FcγRIIa3-EGFP was then generated by the insertion of 57 nucleotides (19-amino acid) into the FcγRIIa1-EGFP plasmid using Phusion Flash polymerase (ThermoFisher) and primers atgggagagaccctccctgagaaaccaGCCAATTCCACTGATCCTGTGAAGG and ttccctgcactcagggtctcctgagagagcTGAAATCCGCTTTTTCCTGCAGTAG. Plasmid DNAs were introduced into RBL-2H3 cells by electroporation (Amaxa) and cells selected in DMEM containing glutamine and 5% heat-inactivated FBS, 0.4 mg/mL Geneticin (Life Technologies).

### Antibody Reagents

The use of non-blocking agonistic anti-FcγRIIa mAb 8.2, the blocking IV.3 mAb, and polyclonal rabbit anti-FcγRIIa ectodomain anti-sera have been previously described (24). Biotin-conjugated Fab fragments of IV.3 and F(ab′)_2_ fragments of mAb 8.2 were generated as described (26).

### Flow Cytometry Analysis of IgG Complex Binding and Receptor Expression

Complexes of human IgG subclasses were generated as previously described (24, 27) using purified myeloma IgG-subclasses (Sigma-Aldrich) and F(ab′)_2_ fragments of anti-human F(ab′)_2_. Briefly, IgG subclasses were incubated with phycoerythrin (PE)-conjugated F(ab′)_2_ anti-human IgG F(ab′)_2_-specific goat antiserum [anti-F(ab′)_2_-PE] (Jackson ImmunoResearch Laboratories) at a 2:1 ratio for 30 min (37°C) and 10 min (4°C).

The binding of these IgG-subclass complexes by the Fc receptors was then determined (24). Briefly, IgG:anti-F(ab′)_2_-PE complexes at the indicated concentrations were incubated with cells (1.2 × 10^5^) in 50 µL for 1 h (4°C), then washed, and resuspended in 200 µL PBS/0.5% BSA. Background binding controls included nonspecific binding of IgG to untransfected, parental IIA1.6 cells.

FcγR expression on transfected cells was quantitated by flow cytometry using a polyclonal anti-FcγRII antiserum (24). Analyses of 10,000 viable cells were performed on at least three independent experiments.

### Membrane Isolation and FcγR Immunoprecipitation

IIA1.6 FcγR transfected cells (5 × 10^6^) were stimulated with non-blocking agonist mAb 8.2 (20 µg/mL), lysed, then membrane isolated according to manufacturer’s specifications (Qproteome Cell Compartment Kit, Qiagen). Briefly, the lysate was clarified by centrifugation at 10,000 *g* for 10 min (4°C) and receptors immunoprecipitated with human IgG (IVIg) (Intragam, CSL, Parkville, Melbourne, VIC, Australia) coated Sepharose beads for 1 h (4°C). The Sepharose beads were washed and bound proteins analyzed by SDS-PAGE. The proteins were transferred to PVDF membranes using a Turbo-blot. Turbo Blotting System (BioRad Laboratories) and FcγRII detected using rabbit anti-FcγRIIa antiserum followed by anti-rabbit Ig/HRP (DakoCytomation). Band signal intensities were enumerated using image J open source Java application (https://imagej.nih.gov/ij/) of precipitated receptor from unstimulated cells was taken as 100% and the intensities of receptor band signals from later time points adjusted accordingly for each replicate.

### Receptor Membrane Colocalization by Fluorescence Microscopy

RBL-2H3 basophilic leukemia cells (1 × 10^7^cells/mL) expressing FcγR-EGFP fusion protein were stimulated with mAb 8.2 (30 µg/mL), incubated for 1 h on ice, washed, and then incubated at 37°C for the indicated time. Cells were then fixed using 4% paraformaldehyde (Electron Microscopy Sciences) and stained with wheat germ agglutinin (WGA) AlexaFluor-633 conjugate (ThermoFisher) and Hoechst 33258 stain (ThermoFisher).

Cells were imaged in PBS at room temperature using a Nikon A1 + -SI laser scanning confocal microscope equipped with a MadCity Labs piezo Z-drive, galvano scanner, 405, 488, 561, and 640 nm lasers and aPlan Apo 60× oil immersion lens (N.A. 1.4). Images were acquired using Nikon NIS-Elements software and analyzed using the open source Java application ImageJ (https://imagej.nih.gov/ij/). Receptor–EGFP colocalization with membrane WGA-AlexaFluor-633 was calculated *via* Pearson’s correlation coefficient using ImageJ plugin JACOP (28) and normalized for resting receptor levels and the fold change in expression calculated.

Sub-diffraction imaging of receptor localization was performed using Structured-Illumination Microscopy (SIM) (29). This technique enables direct comparison between confocal and super-resolution microscopy with no additional sample preparation. The super-resolution images were collected using a Nikon N-SIM microscope equipped with 488, 561, and 640 nm lasers, Andor iXON DU897 EM-CCD camera and a 100× oil immersion lens (N.A. 1.49). The *z*-series was acquired and analyzed as above.

### Calcium Mobilization

IIA1.6 cells were suspended (5 × 10^6^ cells/ml) in calcium release buffer (1×Hanks salts, 10 mM HEPES, 16.7 mM NaHCO_3_, 5.5 mM glucose, 1.8 mM CaCl_2_, and 0.75 mM MgSO_4_, 2.5 mM probenacid, pH7.4.) and incubated at 37°C for 120 min in the presence of 1 µM Fura-2 (ThermoFisher). Cells were subsequently washed then resuspended at 4 × 10^5^ cell/well and stimulated with either FcγRIIa mAb 8.2 or positive control anti-mouse Ig (20 µg/mL) and calcium mobilization determined by ratiometric (340/380 nm) analysis using a FlexStation 3 system (Molecular Devices).

### IgG-Dependent β-Hexosaminidase Degranulation Assay

β-hexosaminidase release was measured colorimetrically, essentially as previously described (30, 31). Briefly, 1.4 × 10^5^ RBL-2H3 cells were resuspended in CD hybridoma media (supplemented with 4 mM glutamine) and mouse IgE anti-TNP mAb (1/2,000 of ascites fluid) added to each well of a 96-well flat-bottom plate and incubated overnight (37°C). Cells were resuspended and stimulated with varying concentrations of anti-FcγRII mAb 8.2 (100–13.35 µg/ml) or TNP:Bovine Serum Albumin (TNP: BSA) (400–0.1 ng/mL) added to IgE positive wells. Cells were stimulated for 30 min (37°C).

Cell supernatants were collected, and the pellet lysed using 200 µL 0.1% Triton X-100 in Tyrodes buffer. Cell supernatants and pellet lysates were assayed for β-hexosaminidase by incubation with 4 mM *p*-nitrophenyl-*N*-acetyl-β-D-glucosaminide (Sigma-Aldrich) for 2 h (37°C) and assay stopped by addition of 0.4 M glycine (pH 10.7) and absorbance measured at 405 nm. The percentage degranulation was calculated by subtracting the spontaneous release from all supernatant OD values and dividing this by those obtained from total cellular β-hexosaminidase quantification.

### Genomic DNA Sequencing

Genomic DNA was isolated from post-Ficoll granulocytes using GenElute Genomic DNA Miniprep (Sigma-Aldrich) and the region of interest PCR amplified using AmpliTaq (LifeTech). *FCGR2A* specific primers (forward, 5′-TGGACTAGCCCTTTTCCAGGT-3′; reverse, 5′-TAGGCCCAGAAA TTAGACTCAGAGT-3′) were used to investigate the intron–exon boundaries of the C1* exon of FcγRIIa and sequences determined using Micromon sequencing services (Melbourne, VIC, Australia).

### Statistics

Results are depicted as mean ± SEM. When applicable, the Student’s *t*-test was used and two-sided test at the 5% significance level (*p* < 0.05). Chi Squared analysis was used for genomic studies with 5% significance level (*p* < 0.05).

## Results

### FcγRIIa3 Sequence in Humans and NHP

**Figure 1:**
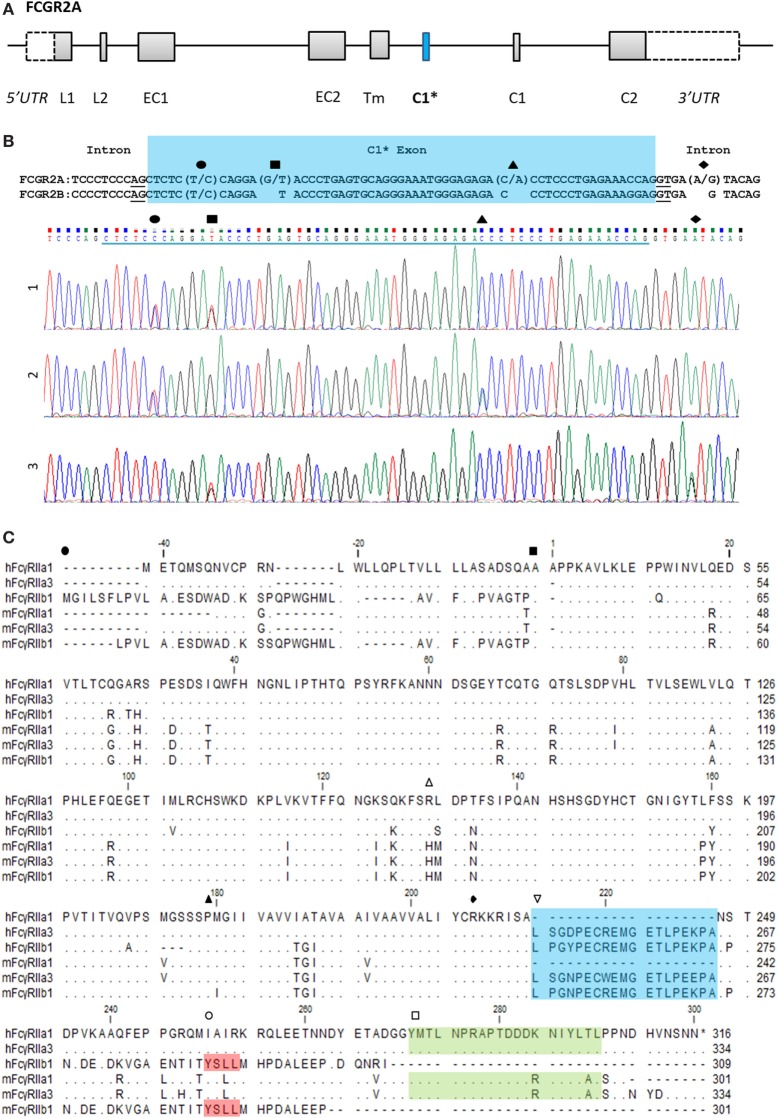
FCGR2A organization and the C1* exon. **(A)** Organization of the *FCGR2A* gene. Exonic regions are represented as boxes and intronic regions as a line. Leader, (EC) extracellular, (Tm) transmembrane, and (C) cytoplasmic tail. **(B)** Alignment of genomic DNA sequence surrounding the C1* exon of *FCGR2A* and *FCGR2B* with single nucleotide polymorphisms and exemplar electropherograms of FcγRIIa3 from three individuals. SNPs are indicated thus: exonic (●) C/T, (■) G/T, and (▲) C/A SNPs which result in proline to serine, aspartic acid to tyrosine, or threonine to asparagine substitution, respectively. The intronic A/G SNP, (◆), in electropherogram 3, reported to promote splicing of C1* exonic sequences results in a unique 19-amino acid insertion in the FcγRIIa3 cytoplasmic tail. **(C)** Amino acid alignment of human (h) and macaque (m) FcγRIIa1, FcγRIIa3, and FcγRIIb1 (32). cDNA sequences were derived from healthy human or macaque PBMC samples. Dot (∙) represents residues matched to human FcγRIIa1 sequence; dash (-) represents a gap in sequence alignment. The N-terminal end of different FcγR structural domains are indicated: (●) leader sequence, (■) extracellular domain, (▲) transmembrane region, and (◆) cytoplasmic tail. Regions of interest are denoted with (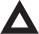) polymorphic 131 residue, (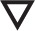) C1 exon (blue), (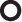) ITIM in FcγRIIb sequence (red), and (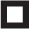) immunoreceptor tyrosine activation motif FcγRIIa (green) regions highlighted.

Novel forms of FcγRIIa were identified during a screen of peripheral blood cells in macaque (*M. nemestrina*) and its expression was confirmed in humans (Figure 1). This receptor, we designate as FcγRIIa3, is identical to canonical FcγRIIa1 except for the inclusion of an additional 57 nucleotides, encoding a 19-amino acid insert in the juxta-membrane region of the cytoplasmic tail (Figure 1C). Sequence analysis indicated FcγRIIa3 arises from the *FCGR2A* gene by the unexpected inclusion of pseudo-exon sequence (C1* exon) previously believed to be untranscribed (8, 20). Moreover, it is transcribed only as human FcγRIIa-R^131^ form and macaque FcγRIIa-H^131^.

The C1* exon-encoded amino acid sequences of human and macaque FcγRIIa3 are highly homologous, with 16 of 19-amino acids identical (Figure 1C). Moreover, they also show near identity (17/19-amino acids) to the unique cytoplasmic sequence of inhibitory FcγRIIb1 (Figure 1C) (32). This sequence in FcγRIIb1 also arises by alternative splicing of the C1 exon of the *FCGR2B* gene and confers delayed ligand-dependent internalization, compared to the FcγRIIb2 isoform, which lacks the C1 exon and which is rapidly internalized (21, 22).

### Interaction Between FcγRIIa3 and Human IgG Subclasses

**Figure 2:**
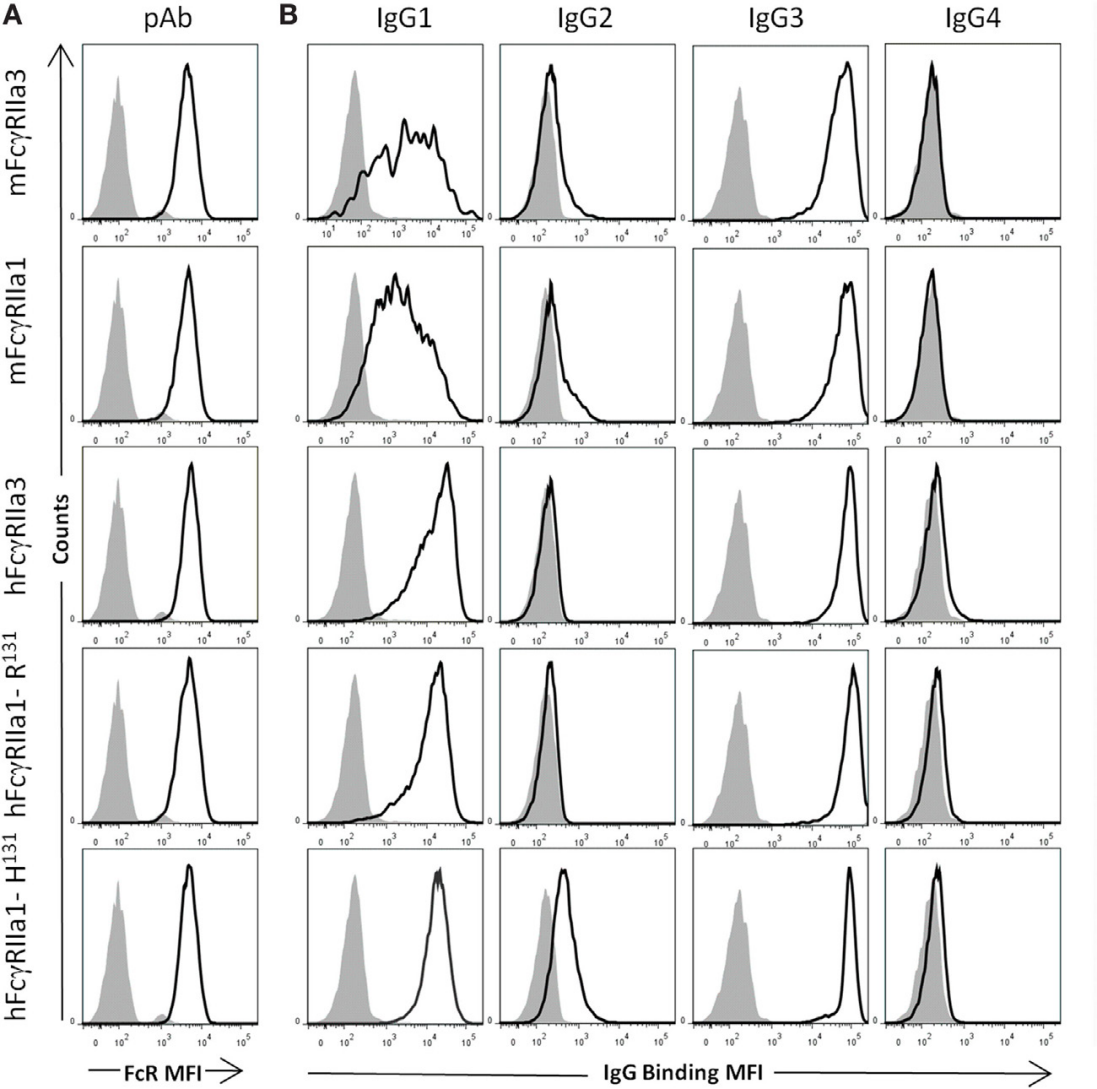
Specificity of FcγRII forms for human IgG subclasses. FcγR expression and IgG binding to IIA1.6 cells expressing macaque mFcγRIIa3-H^131^ or mFcγRIIa1-H^131^ and human allelomorphs FcγRIIa3-R^131^ FcγRIIa1-R^131^, FcγRIIa1-H^131^ expressed on IIA1.6 cells. **(A)** Expression levels of receptor (heavy black open histogram) determined using a rabbit polyclonal anti-FcγRIIa ectodomain antiserum (pAb) (1:100) and PE conjugated anti-rabbit IgG secondary antibody. Background binding of pAb to untransduced IIA1.6 cells (gray filled histogram). **(B)** Binding of human IgG subclass-IgG1, IgG2, IgG3, and IgG4 (10 µg/µL) complexed with PE conjugated human anti-F(ab′)_2_ (5 µg/µL) (heavy black open histogram). Background binding of immune complexes to untransduced IIA1.6 cells (gray filled histogram). For full titration curve see Figure S1 in Supplementary Material.

Comparative analysis of the human IgG-subclass specificity of human and macaque FcγRIIa isoforms was performed by flow cytometry (Figure 2) at multiple IgG concentrations (Figure S1 in Supplementary Material). Similar expression levels of FcγRIIa forms on transfected cells were apparent from the equivalent cell staining using a polyclonal rabbit anti-FcγRIIa antiserum (Figure 2A). The IgG-subclass binding hierarchy was similar in both macaque mFcγRIIa3 and mFcγRIIa1, i.e., IgG3 > IgG1 > IgG2, and IgG4 binding was undetected (Figure 2B). Similarly, the specificity of human FcγRIIa3-R^131^ and hFcγRIIa1-R^131^ was identical: IgG3 > IgG1 > IgG4 >> IgG2 but was distinct from hFcγRIIa1-H^131^ which bound IgG2 (Figure 2B). This was expected due to arginine at position 131, which impairs IgG2 binding to FcγRIIa (33).

Thus, the additional cytoplasmic sequence of either human or macaque FcγRIIa3 does not affect ligand binding as this was indistinguishable from FcγRIIa1 of each species. It is noteworthy that hFcγRIIa variants showed higher binding of IgG1 complexes than equivalent macaque receptors (24).

### Molecular Analysis of Membrane Expression and Internalization Kinetics

**Figure 3:**
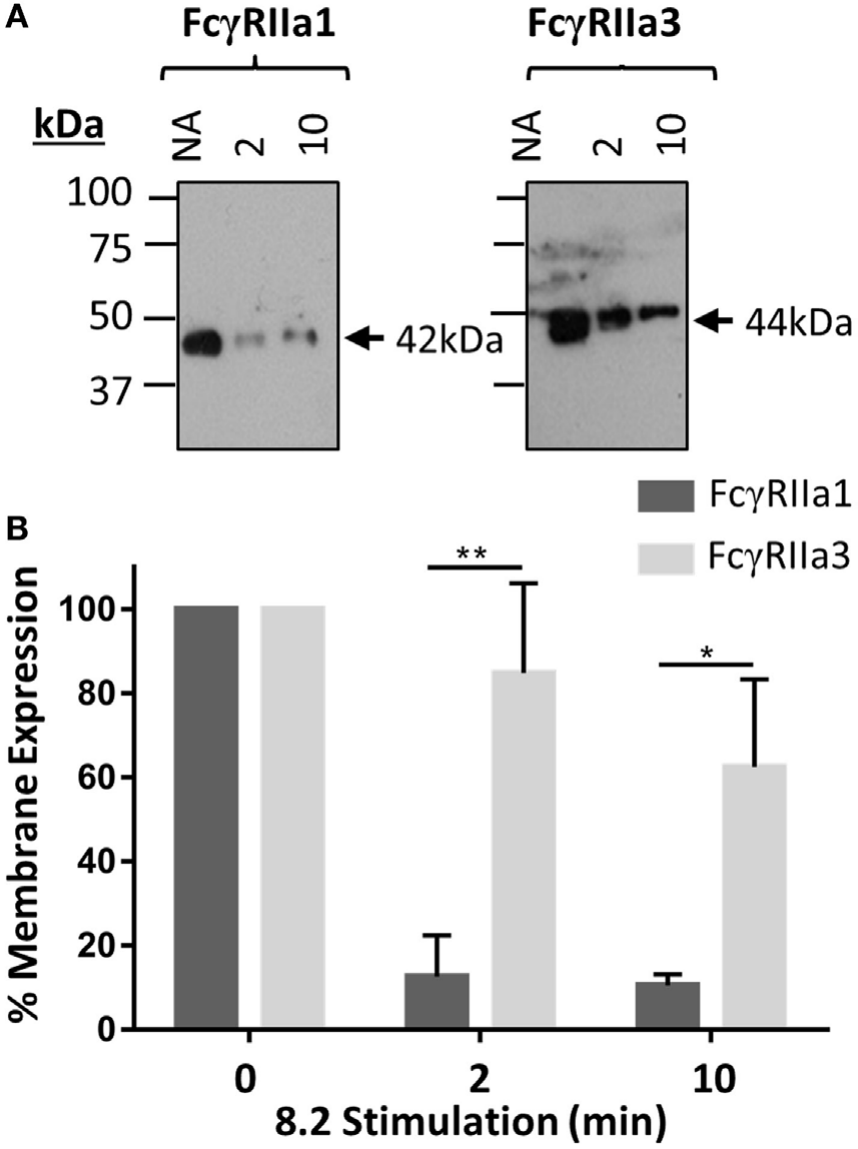
Time course of receptor expression at the plasma membrane following mAb (8.2) stimulation. **(A)** Western blot analysis of FcγRIIa precipitated from cells after stimulation with the non-blocking agonist FcγRIIa mAb 8.2 (20 µg/mL) at 37°C for the indicated time (minutes). Membrane fractions were isolated, receptor immunoprecipitated using IgG-conjugated beads, and subsequently probed using rabbit anti-FcγRIIa anti-sera. Molecular markers and size of FcγRIIa1 (42 kDa) and FcγRIIa3 (44 kDa) are indicated. **(B)** Densitometry of FcγRIIa1 and FcγRIIa3 intensity from three independent Western blot experiments. Intensity of FcγRIIa immunoprecipitation from unstimulated (no addition = NA) cells in each blot is taken as 100% (mean ± SEM, *n* = 3).

The FcγRIIa3 cytoplasmic insert is highly homologous to that of the inhibitory FcγRIIb1, where it impairs receptor endocytosis resulting in prolonged retention of the inhibitory receptor on the cell membrane (21). Thus, a comparison of the cell surface retention of the FcγRIIa3 and FcγRaIIa1 in response to stimulation was assessed by plasma membrane isolation and subsequent immunoprecipitation in transduced IIA1.6 cells (Figure 3). The observed larger size of FcγRIIa3 (44 kDa) compared to FcγRIIa1 (42 kDa) is consistent with the additional 19-amino acids (Figure 3A). Prior to stimulation, both receptors were expressed similarly, however, stimulation lead to considerably different behavior of the receptors (Figure 3A). FcγRIIa1 was rapidly lost from the membrane (Figure 3A) with only 16 and 10% of FcγRIIa1 detectable after 2 or 10 min stimulation, respectively (*p-value* < 0.05) (Figure 3B). By contrast, FcγRIIa3 was mostly retained even after 10 min post-stimulation (Figure 3A) suggesting that the cytoplasmic insert impairs the internalization of FcγRIIa3. Indeed, 85 and 62% of FcγRIIa3 remained at the cell surface after 2 and 10 min, respectively (*p-value* < 0.05) (Figure 3B).

### Analysis of Receptor Distribution by Confocal and Super-Resolution Microscopy

**Figure 4:**
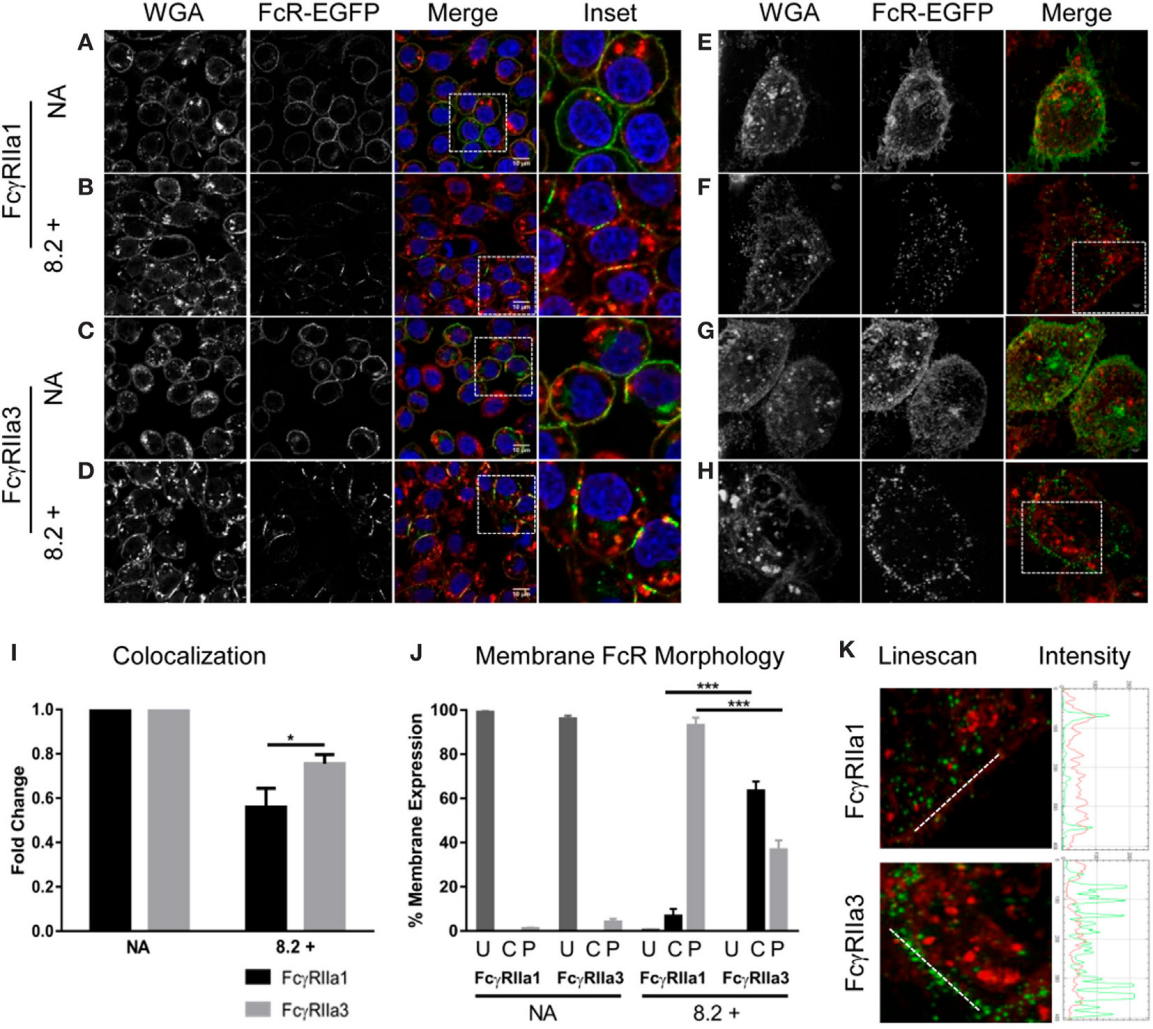
Receptor distribution following stimulation. Panels **(A–D)** are focal plane confocal images of *z*-series (scale bars 10 µm) and panels **(E–H)** are representative 3D maximum intensity projection of N-SIM super resolution images (scale bars 5 µm) of RBL cells expressing FcγRIIa1–EGFP or FcγRIIa3–EGFP (green). Cells were untreated (no addition/NA) [panels **(A,C,E,G)**] or stimulated with mAb 8.2 (30 µg/mL) for 5 min at 37°C [panels **(B,D,F,H)**]. The plasma membrane was stained using wheat germ agglutinin (WGA) AlexaFluor-633 (red) and the nucleus stained using Hoechst 33258 (blue). Cells were imaged in PBS at room temperature using a Nikon A1 + -SI laser scanning confocal or N-SIM microscope and analyzed using the open source Java application ImageJ (see Materials and Methods). Panel **(I)** shows Pearson’s correlation coefficient (*r*) calculated for FcγR and WGA colocalization and normalized for resting state (NA) to represent the fold change in membrane and receptor colocalization (*n* = 3, 10 z-stacks per experiment). Panel **(J)** shows proportion of cells displaying cap or punctate membrane structures determined by blind-counting 100 cells prior to addition (NA) or following 5 min stimulation with mAb 8.2 (mean ± SEM, *n* = 3, 100 cells counted per experiment). Receptor distribution was defined as U = uniform, C = condensed caps, P = punctate morphology (defined in Figure S2 in Supplementary Material). Panel **(K)** Linescan, profiles across membrane segments from stimulated cells as in (panel F and G above). Linescan shown by white line in (left) panels and the corresponding intensity profiles of FcγR (green) and plasma membrane (red) (right).

Since FcγRIIa3 exhibited impaired internalization, the membrane distribution of this receptor variant was further investigated using confocal microscopy (Figures 4A–D) and super-resolution structured-illumination microscopy (N-SIM) (Figures 4E–H) of RBL-2H3 cells expressing EGFP-labeled FcγRIIa forms. In unstimulated cells (“NA” Figures 4A,C,E,G) both FcγRIIa1 and FcγRIIa3 were uniformly expressed at the plasma membrane. However, differences were observed following agonist mAb 8.2 stimulation for 5 min (Figures 4B,D,F,H) where FcγRIIa1 expression at the plasma membrane was decreased compared to FcγRIIa3. These differences in receptor (green) expression and colocalization with plasma membrane/WGA (red) were evaluated and quantified (Figure 4I) using a *z*-series of confocal microscopy images and analyzed by normalizing Pearson’s correlation coefficient (*r*) values (JACOP, ImageJ 28) for resting state. Following activation, the colocalization of FcγRIIa1 (green) with the WGA (red) (56%) was significantly less than for FcγRIIa3 (75%) (*p-value* < 0.05).

Receptor distribution in the membrane was also evaluated by blind-counting 100 cells from representative confocal fields of each experimental treatment group. Each cell was evaluated for receptor distribution (defined in Figure S2 in Supplementary Material) as being of uniform fluorescence distribution or condensed linear cap-like extended fluorescence or punctate fluorescence (Figure 4J). Prior to stimulation, both FcγRIIa1-EGFP and FcγRIIa3-EGFP were uniformly distributed as expected (“NA” Figures 4A,C, and shown in Figure 4J, column “U”) with few condensed cap formation (Figure 4J, column “C” or punctuate structures) (Figure 4J, column “P”). After 5 min stimulation (“8.2+” Figures 4B,D,J) neither receptor was uniformly distributed with clear differences in distribution apparent, particularly the coalescence of FcγRIIa3 into condensed linear cap-like fluorescence (Figures 4D,J; Figure S2 in Supplementary Material). Sixty-three percent (63%) of FcγRIIa3 expressing cells formed these condensed, linear cap-like structures, which were absent from FcγRIIa1-expressing cells where fewer than 6.67% cells were observed with these structures (*p-value* = 0.0005) (Figure 4D). Conversely, almost all of FcγRIIa1-EGFP cells (93%) showed small discrete punctate structures which was significantly higher (*p-value* = 0.0005) than FcγRIIa3-EGFP cells (37%) (Figure 4D).

These morphological observations were then confirmed using N-SIM super-resolution images (Figures 4E–H). Intensity profiles (Figure 4K, red or green trace) were obtained from linescans (Metamorph) (white line) across similar membrane regions (Figure 4K, red trace) and indicated that, the FcγRIIa3 signal (Figure 4K, green trace) was more intense following stimulation than FcγRIIa1 (Figure 4K). Indeed, at this higher resolution of N-SIM, it was apparent that the extended linear cap-like fluorescence of FcγRIIa3 (Figure 4H) was organized as a linear arrangement of punctate elements which was evident in the series of intense peaks in the FcγRIIa3 intensity plots but which were absent from the FcγRIIa1 plots (Figure 4K), while the WGA (red) intensity did not change (Figure 4K).

Thus, quantitative analysis of the receptor colocalization with plasma membrane and the observed morphological differences following receptor ligation demonstrate the two receptor splice variants have distinct behavior in the membrane following ligation. The prolonged membrane retention may also be associated with altered cell activation.

### Increased Calcium Mobilization in IIA1.6 FcγRIIa3 Transfectants

**Figure 5:**
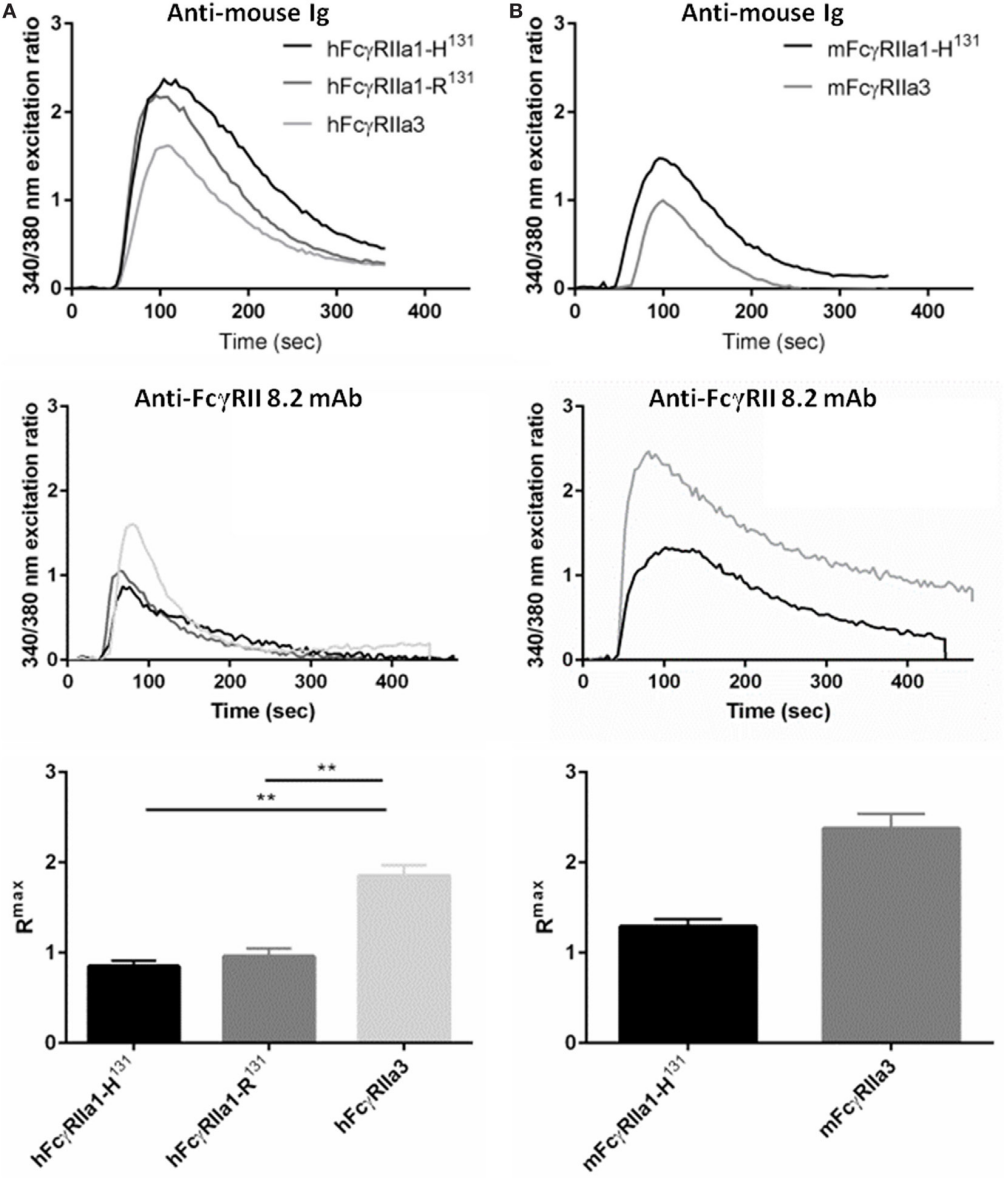
FcγRIIa3 induced greater calcium mobilization. Calcium mobilization in IIA1.6 cells expressing **(A)** human and **(B)** macaque FcγRIIa3 or FcγRIIa1 allelic variants. Cells loaded with fura-2/AM were stimulated at 37°C with agonistic anti-receptor mAb 8.2 or anti-Ig agonist (10 µg/mL), and response measured for 500 s. Representative 340/380 nm excitation ratios indicative of calcium mobilization with background subtracted. R^max^ values were compared to determine peak calcium mobilization levels (mean ± SEM, *n* = 3).

FcR ITAM phosphorylation results in intracellular calcium (Ca^2+^) mobilization (34). Thus, the human and macaque FcγRIIa splice variants in IIA1.6 cells were tested for their capacity to induce a Ca^2+^ response to the anti-FcγRIIa mAb 8.2 and control anti-mouse Ig agonists. hFcγRIIa3 stimulation resulted in significantly higher R^max^ compared to activation *via* hFcγRIIa1-R^131^ (*p-value* = 0.0037) or hFcγRIIa1-H^131^ (*p-value* = 0.0017) (Figure 5A). In the macaque, ligation of the mFcγRIIa3 induced significantly higher R^max^ in comparison to mFcγRIIa1 (*p-value* = 0.004) (Figure 5B). The enhanced Ca^2+^ response was not related to allotypic high/low-responder forms, hFcγRIIa1-H^131^ and hFcγRIIa1-R^131^ which induced similar R^max^ values and Ca^2+^ mobilization curves (*p-value* = 0.39) (Figure 5A). Furthermore, differences in FcγR-driven Ca^2+^ mobilization were not due to differences in inherent capacity of cells as stimulation *via* the endogenous BCR was similar for all cell lines (Figure 5).

### FcγRIIa3 Transfected Mast Cells Have Increased IgG-Dependent Degranulation

**Figure 6:**
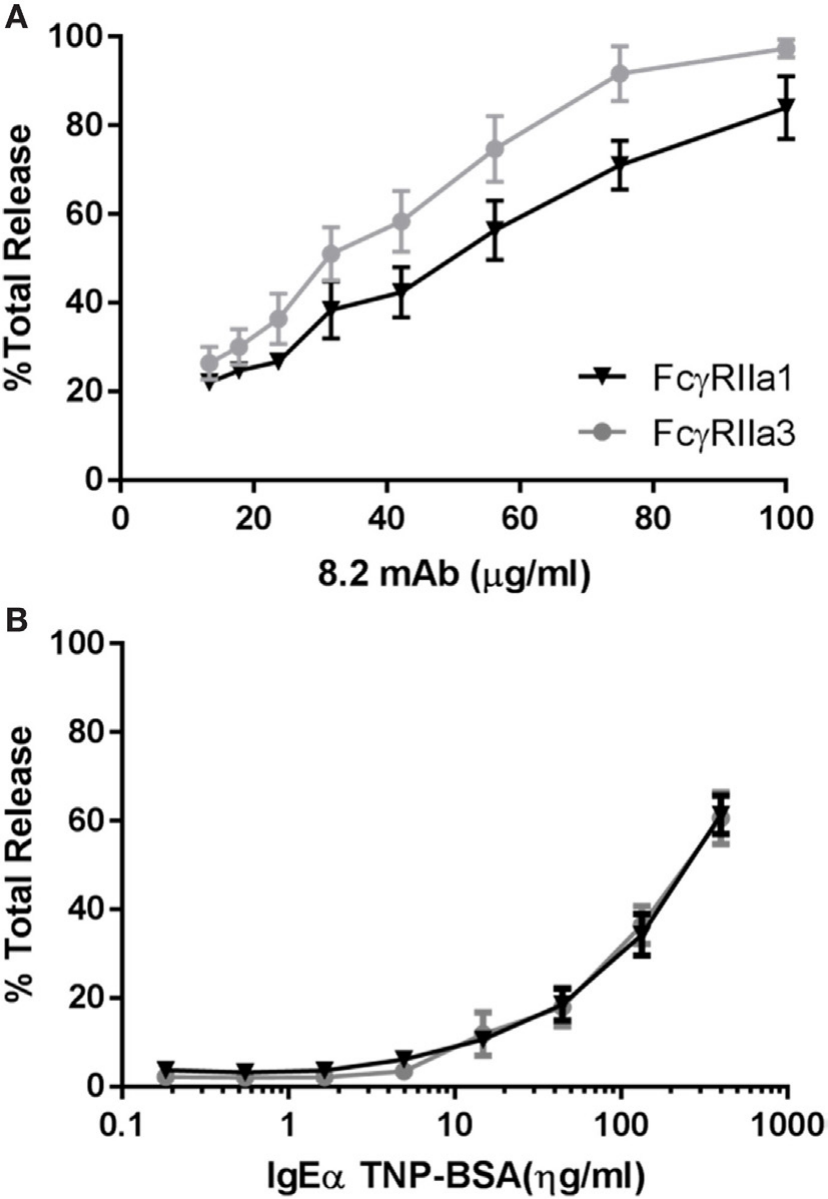
Increased levels of β-hexosaminidase release. RBL cells expressing human allelomorphs FcγRIIa1-R^131^ (▾) or FcγRIIa3 (●) as indicated. Transfectants were stimulated with **(A)** anti-FcγRIIa mAb 8.2 agonist (100–13.35 µg/mL) and **(B)** IgE anti-TNP/TNP:BSA (0.1–400 ng/mL) for 30 min at 37°C. Supernatant was then incubated with β-hexosaminidase substrate (4 mM p-NAG) and the percentage normalized cell total OD plotted as a dose response curve (mean ± SEM, *n* = 3). Student *t*-test, *p-value* = 0.0005.

The increased Ca^2+^ mobilization and longer membrane retention suggested that FcγRIIa3 activation may also alter activation of cell function. RBL basophilic cells are widely used in studies of FcγRII and FcεRI activation of cells by measuring degranulation-related release of β-hexosaminidase (35). Thus RBL-2H3 cells that expressed FcγRIIa3 or FcγRIIa1 were stimulated with mAb 8.2. As expected FcγRIIa1 induced β-hexosaminidase over the range of agonist concentrations but FcγRIIa3 induced significantly increased β-hexosaminidase release in FcγRIIa3 (*p-value* = 0.005). This difference was most apparent at the higher agonist concentrations (Figure 6A). As expected, control stimulation *via* the IgE receptor (FcεRI) induced identical β-hexosaminidase release in both FcγRII-expressing cell lines (Figure 6B).

### Evaluation of FcγRIIa3 SNP in Immune Deficiency and SLE

Genomic DNA of 224 subjects was analyzed for genetic polymorphisms in and around exon C1* (Table 1; Figure 1B). Analysis was conducted on 78 patients with a primary antibody deficiency who were grouped as follows: 46 CVID; 15 IgG-deficiency (hypogammaglobulinemia or specific antibody or IgG-subclass deficiency); 11 sIgAD and 6 XLA; and then 91 SLE patients and 55 healthy subjects, none of which had adverse reactions to treatment. Sequencing revealed an intronic SNP and three additional polymorphic positions within exon C1*. The intronic SNP *FCGR2A^g.6,413A^*^>^*^G^* (nucleotide numbering from NG_012066.2) has been reported in a separate study as *FCGR2A^c.742+871A^*^>^*^G^* where it was associated with CVID and adverse responses to IVIg therapy and with several other immune disorders (19). In our study this SNP (bolded in Table 1), was found only in one of the 448 genomes analyzed, in a patient with IgA-deficiency, vitiligo, atrophic gastritis, and autoimmune epididymo-orchitis. Surprisingly, it was not found in the CVID group using similar numbers as the previously reported study, nor was it apparent in other immune deficiencies that we investigated nor in SLE patients, a disease where other *FCGR2A* SNP disease-associations have been reported reviewed in Ref. (1).

**Table 1:** Analysis of intronic and exonic SNP associated with *FCGR2A* C1 exon^a^.

	Controls, no. (%)	Common variable immunodeficiency (CVID), no. (%)	IgG deficiency, no. (%)	sIgAD, no. (%)	XLA, no. (%)	Systemic lupus erythematosus (SLE), no. (%)
		
	*n* = 55	*n* = 46	*n* = 15	*n* = 11	*n* = 6	*n* = 91
*****FCGR2A^g.6,357G>T^***(serine > proline)**						

	*Genotype frequency*					

CC	18 (33.3)	15 (32.6)	5 (35.7)	4 (36.4)	2 (33.3)	38 (41.8)
CT	28 (51.9)	16 (34.8)	6 (42.9)	5 (45.5)	4 (66.7)	40 (44.0)
TT	8 (14.8)	15 (32.6)	3 (21.4)	2 (18.2)	0 (0.0)	13 (14.3)
*P* value	–	0.0793	0.7791	0.9204	0.5738	0.5828

	*Allele frequency*					

C	64 (59.3)	46 (50.0)	16 (57.1)	13 (59.2)	8 (66.7)	116 (63.7)
T	44 (40.7)	46 (50.0)	12 (42.9)	9 (40.9)	4 (33.3)	66 (36.3)
*P* value	–	0.1896	0.8393	0.9883	0.6193	0.4475

*****FCGR2A^g.6,363G>T^***(aspartic acid > tyrosine)**						

	*Genotype frequency*					

GG	42 (76.4)	29 (63.0)	14 (93.3)	7 (70.0)	4 (66.7)	64 (70.3)
GT	11 (20.0)	14 (30.4)	1 (6.7)	3 (30.0)	2 (33.3)	21 (23.1)
TT	2 (3.6)	3 (6.5)	0 (0.0)	0 (0.0)	0 (0.0)	6 (6.6)
*P* value	–	0.3404	0.3342	0.6697	0.6918	0.6282

	*Allele frequency*					

G	95 (86.4)	72 (78.3)	29 (96.7)	17 (84.0)	10 (83.3)	149 (81.9)
T	15 (13.6)	20 (21.7)	1 (3.3)	3 (15.0)	2 (16.7)	33 (18.1)
*P* value	–	0.7691	0.1159	0.1624	0.7735	0.3152

*****FCGR2A^g.6,391C>A^***(threonine > asparagine)**						

	*Genotype frequency*					
CC	53 (96.4)	45 (97.8)	13 (86.7)	11 (100.0)	5 (83.3)	90 (99.9)
CA	2 (3.6)	1 (2.2)	2 (13.3)	0 (0.0)	1 (16.7)	1(1.1)
AA	0 (0.0)	0 (0.0)	0 (0.0)	0 (0.0)	0 (0.0)	0 (0.0)
*P* value	–	0.9386	0.1515	0.6423	0.1611	0.2950

	*Allele frequency*					

C	108 (98.2)	91 (98.9)	28 (93.3)	22 (100.0)	11 (91.7)	181 (99.5)
A	2 (1.8)	1 (1.1)	2 (6.7)	0 (0.0)	1 (8.3)	1 (0.5)
*P* value	–	0.6223	0.1862	0.5079	0.1921	0.2644

*****FCGR2A^g.64131A>G^***(Intronic splice donor)**						

	*Genotype frequency*					

AA	55 (100.0)	46 (100.0)	15 (100.)	**10 (90.9)**	6 (100.0)	91 (100.0)
AG	0 (0.0)	0 (0.0)	0 (0.0)	**1 (9.1)**	0 (0.0)	0 (0.0)
GG	0 (0.0)	0 (0.0)	0 (0.0)	**0 (0.0)**	0 (0.0)	0 (0.0)
*P* value	–	N/A	N/A	**0.0242**	N/A	N/A

	*Allele frequency*					

A	110 (100.0)	92 (100.0)	30 (100.0)	**21 (95.5)**	12 (100.0)	182 (100.0)
G	0 (0.0)	0 (0.0)	0 (0.0)	**1 (4.5)**	0 (0.0)	0 (0.0)
*P* value	–	N/A	N/A	0.0248*	N/A	N/A

*^a^Patient groups include: CVID; IgG deficiency (hypogammaglobulinaemia or specific Ig or IgG deficiency); sIgAD (selective IgA-deficiency) or XLA (X-linked agammaglobulinaemia); and SLE. Nucleotide numbering from NG_012066*.

*The bolded text the sIgAD group highlights the only patient with the intronic splice donor SNP*.

Additional SNPs were identified within the exon. Two novel SNPs *FCGR2A^g.6,363G^*^>^*^T^* encodes either aspartic acid or tyrosine and *FCGR2A^g.6,391C^*^>^*^A^* encodes threonine or asparagine (Table 1). Neither showed significant associations with immunodeficiency or SLE. A third SNP, *FCGR2A^g.6,357G^*^>^*^T^* results in a serine to proline substitution (Table 1), is known in the *FCGR2A* gene and a similar SNP has been identified in *FCGR2B* and *FCGR2C* but no disease association was apparent.

## Discussion

Herein, we describe the role and genetics of a novel form of FcγRIIa. This FcγRIIa3 contains a highly conserved insertion in its cytoplasmic membrane proximal region in both humans and NHP. In NHP FcγRIIa3 of different macaque species, pigtailed (Figure 1) and cynomolgus (XP_015307808.1 and XP_015307801.1) contain a nearly identical insertion that is also highly related to their human equivalents, suggesting that the FcγRIIa3 is evolutionarily conserved in higher primates.

The 19-amino acid insert of human FcγRIIa3 arises from the inclusion of the C1* exon that has historically been regarded as an untranscribed evolutionary remnant sequence (8, 20). Genomic DNA sequencing of healthy donors and immune deficient or SLE patients identified several novel SNPs within this exon, but importantly, also identified the intronic *FCGR2A^g.6,413A^*^>^*^G^* (also known as *FCGR2A^c.742+871A^*^>^*^G^*) which facilitates splicing to retain the C1* exon (19). Moreover, this SNP is clinically important as it is reportedly associated with pathological adverse responses to IVIg therapy in some CVID (19) patients expressing FcγRIIa3 where immune complex formation *in vivo*, lead to potent cell activation and anaphylaxis.

Our functional analysis, and genetic analyses of patients, was directed at establishing how the presence of the 19-amino acid cytoplasmic insert affected receptor function and determining the frequency of the SNP distribution in our patient groups. Binding studies revealed that the cytoplasmic insert did not affect ligand specificity. Both human and macaque FcγRIIa3 bound human IgG1, IgG3, and IgG4 subclasses with identical specificity of the FcγRIIa1 of each species (24, 33, 36).

Although human IgG-subclass binding did not differ, the presence of the insert altered FcγRIIa function. Organization of FcγRIIa3 on the cell was distinct from the canonical FcγRIIa1. Both biochemical and fluorescence microscopy revealed altered expression and distribution of liganded receptor. FcγRIIa3 expression was prolonged at the plasma membrane following receptor stimulation in contrast to the rapid loss of FcγRIIa1. Microscopy also showed retention of FcγRIIa3 in the membrane but additionally visualized FcγRIIa3 in condensed cap-like clusters and some punctate structures. In contrast, FcγRIIa1 predominantly formed punctate structures with few caps observed.

The presence of activated receptor: agonist complexes for longer at the plasma membrane may contribute to the enhanced activating signaling by the FcγRIIa3 variant. This enhanced signaling included increased Ca^2+^ mobilization and resulted in increased cellular activation and degranulation.

Although the cellular responses driven *via* the ITAM containing FcγRIIa1/FcγRIIa3 forms and the ITIM-containing FcγRIIb1/FcγRIIb2 are different, there are aspects of their biology that are clearly analogous. The juxta-membrane cytoplasmic insert of FcγRIIa3 and FcγRIIb1 are highly homologous and are similarly derived by alternative mRNA splicing of the C1* or C1 exon, from the respective genes. Its inclusion in the receptors confers similar properties on FcγRIIa3 and FcγRIIb1 in the cell membrane. Indeed the homologous C1* insert is necessary for the condensed cap-like organization of FcγRIIa3 (Figure 4) which is similar to the liganded caps of FcγRIIb1 (21), but are quite distinct from the rapidly internalizing FcγRIIa1 and FcγRIIb2, both of which lack the C1* or C1 insert and do not form the cap-like structures (21, 37).

The C1* exon that encodes the FcγRIIa3 insertion had been previously assumed to be a pseudo-exon (20). More recently, though a C1* intronic SNP *FCGR2A^c.742+871A^*^>^*^G^* (19), identical to our *FCGR2A^g.6,413A^*^>^*^G^*, was identified and generates a mRNA splice donor site resulting in high levels of receptor expression but is also rare in healthy individuals (1/287). In that study, the SNP was considerably more frequent in patients with CVID (3/53 patients) and with those patients that had anaphylactic reactions to their IVIg therapy. In our study, the SNP was also rare but notably was not found in our CVID patients, neither the majority of primary antibody deficient patients nor any SLE patient. Indeed, only a single individual, heterozygous for the SNP, was detected among all 224 individuals. It is worth noting that our index patient has not received IVIg treatment but is presumably at risk of adverse reaction to IVIg. The reason for the differences between our studies and those of the previous study (19) are not clear but ethnological differences may also contribute to difference in allele frequency. Importantly, no adverse reactions to IVIg therapy have been noted in our patients which are an additional important distinction between our data and the previous study.

None-the-less the presence of the SNP and expression of FcγRIIa3 may contribute to antibody-dependant proinflammatory pathologies in autoimmune disease or adverse response in therapeutic settings or autoimmune diseases. Indeed, its prolonged membrane retention after ligation and its capacity to induce enhanced or exaggerated cell activation is consistent with the increased sensitivity of anaphylactic CVID patient neutrophils to stimulation by small immune complexes as reported by others (19).

Thus, even though relatively rare, the expression of FcγRIIa3 may under normal circumstances offer an advantage of a heightened protective response under or, conversely, may contribute adversely to exacerbated inflammation in antibody-mediated pathology or to adverse reactions to antibody therapy.

## Ethics Statement

The collection and analysis of human peripheral blood samples was carried out in accordance with the recommendations of National Statement on Ethical Conduct in Human Research of the Australian National Health and Medical Research Council. The protocol was approved by the Human research ethics committees of the administering institutions: Monash University (2015-0344; 2016-0289; 14262A; and 15510L), Alfred Health (497/11 and 109/15), Melbourne Health (2009.162), and Walter and Eliza Hall Institute (WEHI, 10/02). All subjects gave written informed consent in accordance with the Declaration of Helsinki. Peripheral blood of macaques was obtained in accordance with Australian National Health and Medical Research Council, Australian Code for the Care and Use of Animals for Scientific Purposes. The project was approved by the Institutional Animal Research Ethics Committees of the University of Melbourne and Commonwealth Scientific and Industrial Research Organization Animal Health.

## Author Contributions

JA: designed and performed experiments, analyzed data, and wrote the manuscript. PMH and BW: designed experiments, analyzed data, and wrote the manuscript. HT, PT, CP, BK, AC, PA, GM, and MZ: assisted with experiments, data analysis, and manuscript revision. SK, MZ, AH, RK, CS, VB, and PH: provided samples for genetic analysis, data analysis, and manuscript revision.

## Conflict of Interest Statement

The authors declare that the research was conducted in the absence of any commercial or financial relationships that could be construed as a potential conflict of interest.
