# Supplementary material for: The Rare Anaphylaxis-Associated FcγRIIa3 Exhibits Distinct Characteristics From the Canonical FcγRIIa1
Source: Front Immunol. 2018 Aug 20;9:1809. doi: 10.3389/fimmu.2018.01809 (PMC6109644; doi:10.3389/fimmu.2018.01809)
Supplement: Supplementary file 1 [file data_sheet_1.pdf]

SUPPLEMENTARY FIGURES

Figure S1: Titration of human IgG subclass binding to FcγRIIa forms. IIA1.6 cells expressing macaque allelomorphs mFcγRIIa3-R^{131}, mFcγRIIa1-R^{131} and human allelomorphs FcγRIIa3-R^{131}, FcγRIIa1-R^{131} and FcγRIIa1-H^{131} as indicated. Two-fold dilutions (10-0.3μg/μL IgG) (represented by) of IgG: F(ab`)$_2$-PE complexes were titrated and binding determined by flow cytometry (mean ±SEM, n=3). Note differences in y-axis. See Figure 1 for flow cytometric fluorescence profiles.

Figure S2: Membrane receptor expression. Exemplar images of receptor distributions following stimulation of receptor expressing cells used for blind counting in Figure 4. Cells were imaged in PBS at room temperature using a Nikon A1+-SI laser scanning confocal microscope and analysed using the open source Java application ImageJ (see Methods for detail). Uniform (evenly dispersed fluorescence); Condensed linear cap-like fluorescence; Punctate (small discrete fluorescence).

SUPPLEMENTARY FIGURES

Supplementary Figure 1.

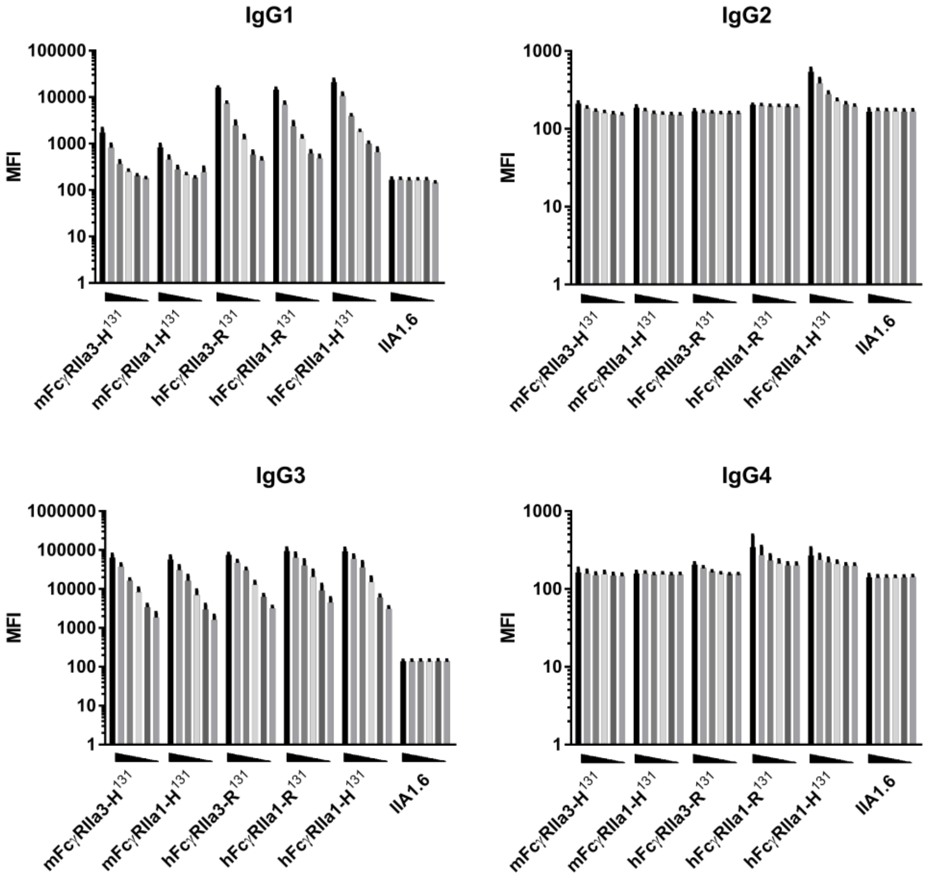

Supplementary Figure S2.

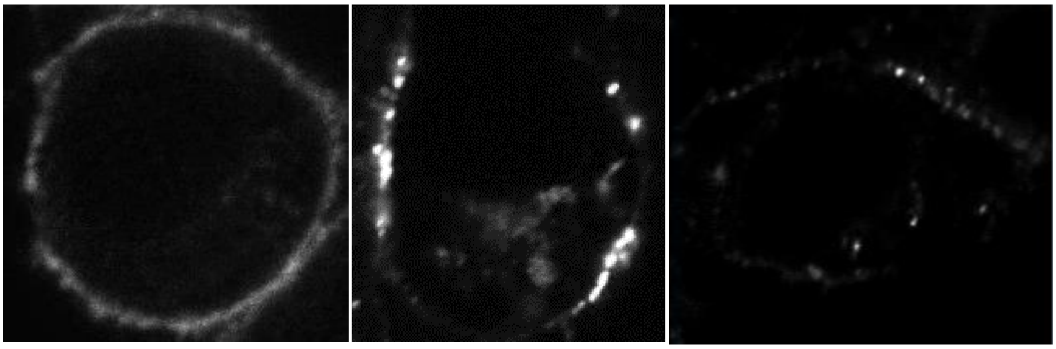

Uniform Condensed Punctate
